# Cu(β-diketonato)_2_ bathochromic shifts from the ultraviolet towards the visible region

**DOI:** 10.1007/s00894-024-06138-1

**Published:** 2024-09-17

**Authors:** Marrigje M. Conradie

**Affiliations:** Chemistry Department, University of the Free State, Bloemfontein, Republic of South Africa

**Keywords:** DSSC, TDDFT, Copper(II), β-Diketone

## Abstract

**Context:**

The DFT-calculated ultraviolet/visible properties of 11 different Cu(β-diketonato)_2_ complexes are presented. The selected β-diketonato ligands on the Cu complex contain none, one or two aromatic rings. The experimentally measured absorbance maxima range of the ultraviolet/visible is observed at 295–390 nm, and the calculated range is 302–425 nm, for the 11 complexes in this study. More aromatic rings on the ligand lead to bathochromic shifts of the experimentally measured absorbance maxima from the ultraviolet towards the visible region. Absorbance maxima of the Cu(β-diketonato)_2_ complexes with no aromatic rings on the ligand are found to be predominantly ligand-to-metal charge transfer excitations, whereas introducing one or two aromatic rings shifts the excitations to predominantly ligand-to-ligand charge transfer.

**Methods:**

DFT calculations were conducted on the neutral molecules with multiplicity 2, using the PBEh1PBE functional and the aug-cc-pVDZ basis set as implemented in the Gaussian 16 package. The selected solvent was acetonitrile, the solvent in which most of the experimental UV/Vis are reported. The molecules were all optimized in the solvent phase, using the IEFPCM. The initial coordinates for the compounds were generated using Chemcraft.

**Highlights:**

TDDFT of 11 different Cu(β-diketonato)_2_ complexes follow the experimental trend.

Aromatic rings on the ligand lead to Bathochromic shifts of UV/Visible spectra.

No aromatic rings on the ligand lead to ligand-to-metal charge transfer excitations.

Aromatic rings on the ligand lead to ligand-to-ligand charge transfer excitations.

**Graphical abstract:**

Bathochromic shifts in eco-friendly Cu(β-diketonato)_2_.

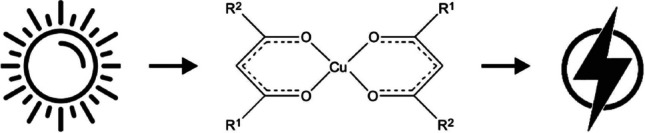

**Supplementary information:**

The online version contains supplementary material available at 10.1007/s00894-024-06138-1.

## Introduction

The increase in the global demand for electricity, propelled by a rising population, is challenging due to the reliance on diminishing fossil fuel resources [1]. Solar energy, particularly through silicon-based solar cells, presents an eco-friendly alternative, though it faces issues like complex manufacturing, hazardous materials and high costs [2]. This has led to the evolution of low-cost dye-sensitized solar cells (DSSCs), which use dyes, electrolytes, photoanodes and counter electrodes to convert sunlight into electricity [3, 4]. DSSCs, a third-generation photovoltaic technology, are sustainable and reduce greenhouse gas emissions, offering energy independence and solutions for remote areas. They efficiently operate under various lighting conditions and utilize dyes that absorb and generate charged particles, categorized into natural, organic metal-free and inorganic metal-containing dyes [5].

**Fig. 1:**
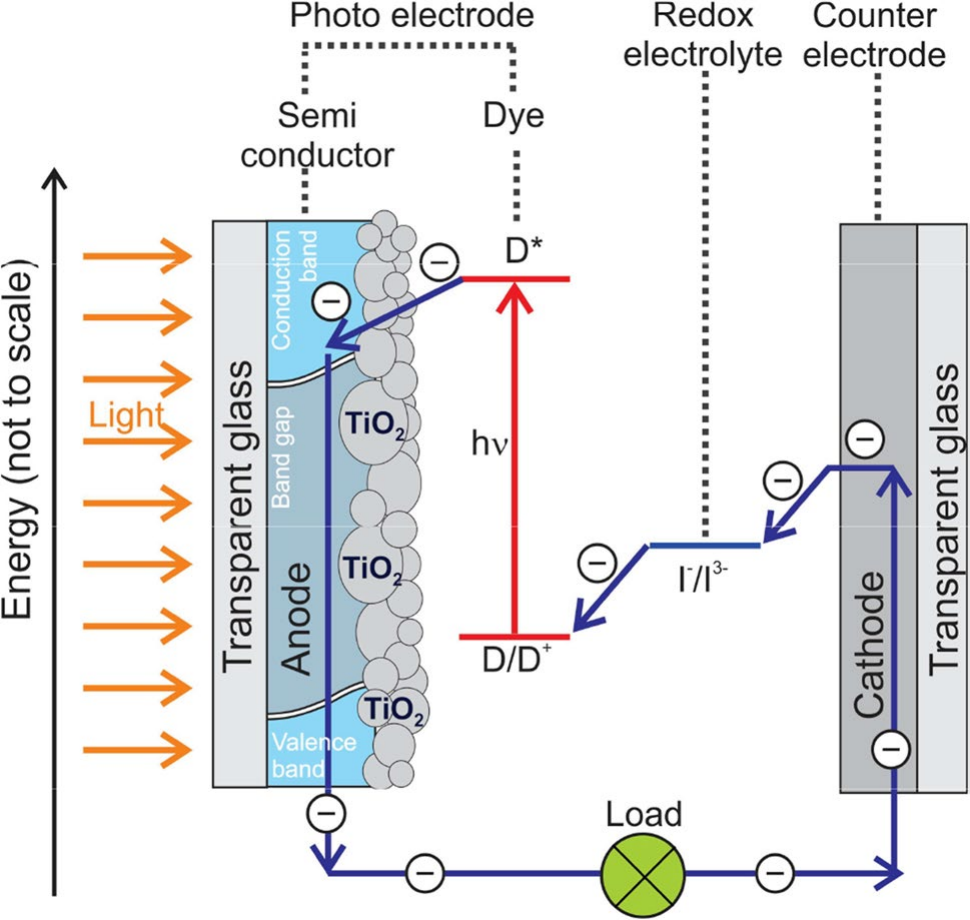
“Diagram showing the basic operation of a DSSC containing a TiO_2_ semi-conductor and an I^−^/I^3^^−^ redox electrolyte.” from reference [6] (open access)

The efficiency of DSSCs relies heavily on the dye’s capability to absorb sunlight. UV/Vis spectroscopy measures the dye’s absorbance across the UV and visible light spectrum, indicating how effectively the dye can harvest light energy. The UV/Vis properties aid in understanding the excitation of electrons within the dye molecules. When light energy is absorbed by the dye in the UV/Vis range, it generates excited electrons, which are then transferred to the semiconductor, initiating the process of electricity generation; see Fig. 1.

**Scheme 1:**
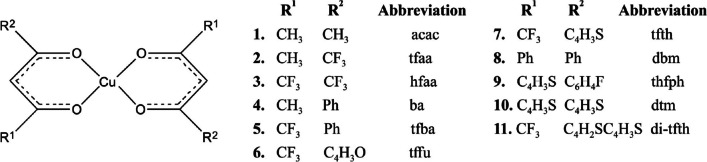
The structure and sequencing of the Cu(β-diketonato)_2_ complexes presented in this study, including abbreviation used for the β-diketonato ligand

Focusing on inorganic metal-containing dyes, copper, a plentiful and non-toxic metal, has emerged as a promising and cost-effective alternative to ruthenium in DSSCs [7, 8]. Various Cu complexes including phenanthroline or bipyridine ligand substituents have been experimentally evaluated [9–15] and examined theoretically [7, 16] as potential dye sensitizers. This study aims to systematically investigate 11 different Cu(β-diketonato)_2_ complexes, as indicated in Scheme 1. The effect of different groups on the β-diketonato ligand on the UV/Vis spectra will be investigated, to establish which substituents will lead to bathochromic (red) shifts. Theoretical insights from this study could contribute to the development and evaluation of high-efficiency dyes for DSSCs, which is the main motivation behind this computational investigation.

## Theoretical calculations

Density functional theory (DFT) calculations were conducted on the neutral molecules with multiplicity 2, using the PBEh1PBE [17] functional and the aug-cc-pVDZ [18, 19] basis set as implemented in the Gaussian 16 package [20]. The selected solvent was acetonitrile, the solvent in which most of the experimental UV/Vis are reported [21, 22]. This functional/basis set combination previously showed the best performance of optimization and TDDFT calculations for related Cu(β-diketonato)_2_ complexes [23]. The molecules were all optimized in the solvent phase, using the integral equation formalism polarizable continuum model (IEFPCM) [24, 25]. The initial coordinates for the compounds were generated using Chemcraft [26].

Several properties important for the effective functioning of DSSC can be calculated theoretically [6] and are calculated here for **(1)**–**(11)** using Eqs. 1–4 below. These properties are determined as outlined in the literature [16] for a dye in a cell having TiO_2_ as a semiconductor and I^−^/I^3−^ as redox electrolyte, as often used when theoretically evaluating dyes for application in DSSCs [27–35].

Light harvesting efficiency (LHE) can be obtained by [36–40]:

1 $$\text{LHE}=1-{10}^{-f}$$

Here, *f* is the TDDFT calculated oscillator strength of the absorption bands.

The excited state lifetime (τ) of the excitation at λ of dyes can be obtained by [8]:

2 $$\tau \left(\text{in }s\right)=\frac{1.499}{f{\left({E}_{\lambda }\right)}^{2}}$$

Here, *E*_λ_ is the calculated transition energy (cm^−1^), and *f* is the calculated oscillator strength of the excited state corresponding to the specified wavelength λ, obtained from TDDFT calculations [23].

The driving force of electron injection (Δ*G*_inject_) and the driving force of dye regeneration (Δ*G*_regenerate_) can be obtained from oxidation potentials by [8, 32]:

3 $$\Delta {G}_{\text{inject}}= {E}_{\text{dye}}^{*}- {E}_{\text{CB}}= {E}_{\text{HOMO}}- {E}_{{\uplambda }_{\text{max}}} - {E}_{\text{CB}}$$

4 $$\Delta {G}_{\text{regenerate}}= \left({E}_{{\text{I}}^{-}/{\text{I}}_{}^{3-}} \right)-\left({E}_{\text{dye}}\right)$$

Here, *E*_dye_ is the oxidation potential of the dye (estimated by ${E}_{\text{HOMO}}$). ${E}_{\text{dye}}^{*}$ is the oxidation potential of the excited dye (estimated by ${E}_{\text{Homo}}- {E}_{{\uplambda }_{\text{max}}}$). *E*_CB_ is the reduction potential of the conduction band edge of TiO_2_ (− 4.0 eV vs vacuum or − 0.5 eV vs NHE [41]). ${E}_{{\uplambda }_{\text{max}}}$ is the electronic vertical transition energy corresponding to λ_max_. ${E}_{{\text{I}}^{-}/{\text{I}}_{}^{3-}}$ is the redox potential of the electrolyte I^−^/I^3−^ redox couple (− 4.8 eV vs vacuum or 0.3 eV vs NHE) [23, 42].

## Results and discussion

### Geometry and electronic structure

**Fig. 2:**
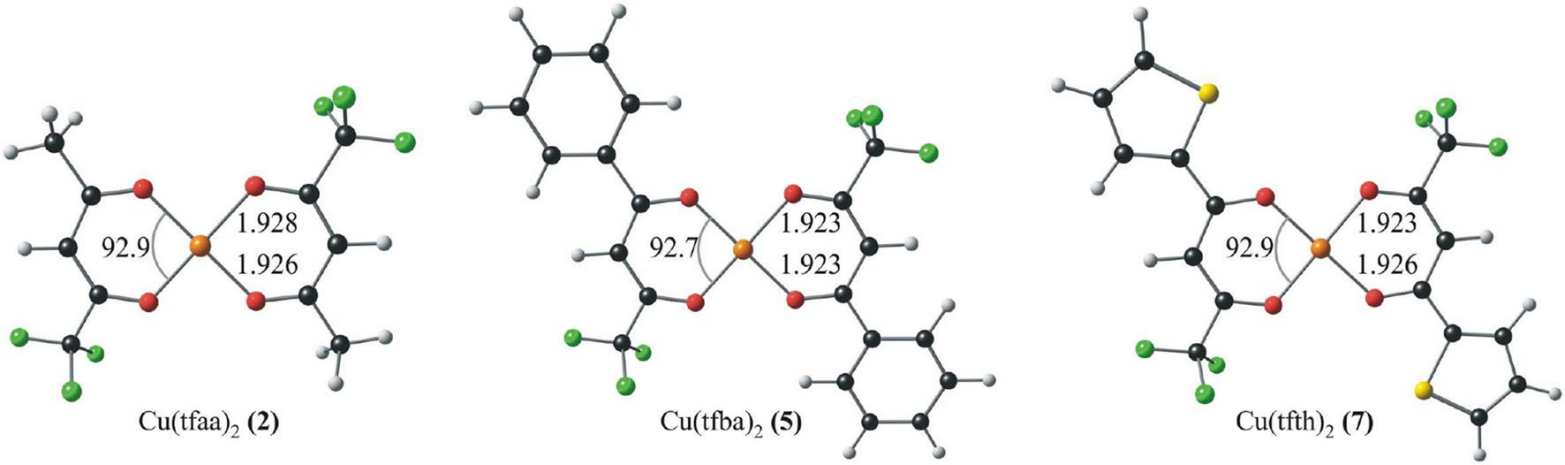
Optimized geometry of selected Cu(β-diketonato)_2_ complexes. Bond lengths (Å) and angles (°) are as illustrated. Atoms are coloured as follows: Cu (orange), C (grey), O (red), F (green), S (yellow) and H (white)

Cu(β-diketonato)_2_ complexes are d^9^ copper(II) with spin ½, and thus, 5 alpha and 4 beta predominantly copper-based occupied molecular orbitals (MOs) under the frontier MOs, with only 1 unoccupied copper-based MO, that is the LUMO in the case of Cu(acac)_2_ [23]. The β-diketones form a square geometry planar around the copper(II) centre in the optimized geometries, as illustrated in Fig. 2.

The molecular structure of the unsymmetrical Cu(β-diketonato)_2_ complexes **(2)**, **(4)**–**(7)**, **(9)** and **(11)**, can theoretically be a *cis*- or a *trans*-isomer. Since only the *trans*-isomers were experimentally isolated [43–45] and the *trans*-isomers have lower energies than *cis*-isomers [22, 43], calculations were performed using only the *trans*-isomers.

### UV/Vis properties

**Fig. 3:**
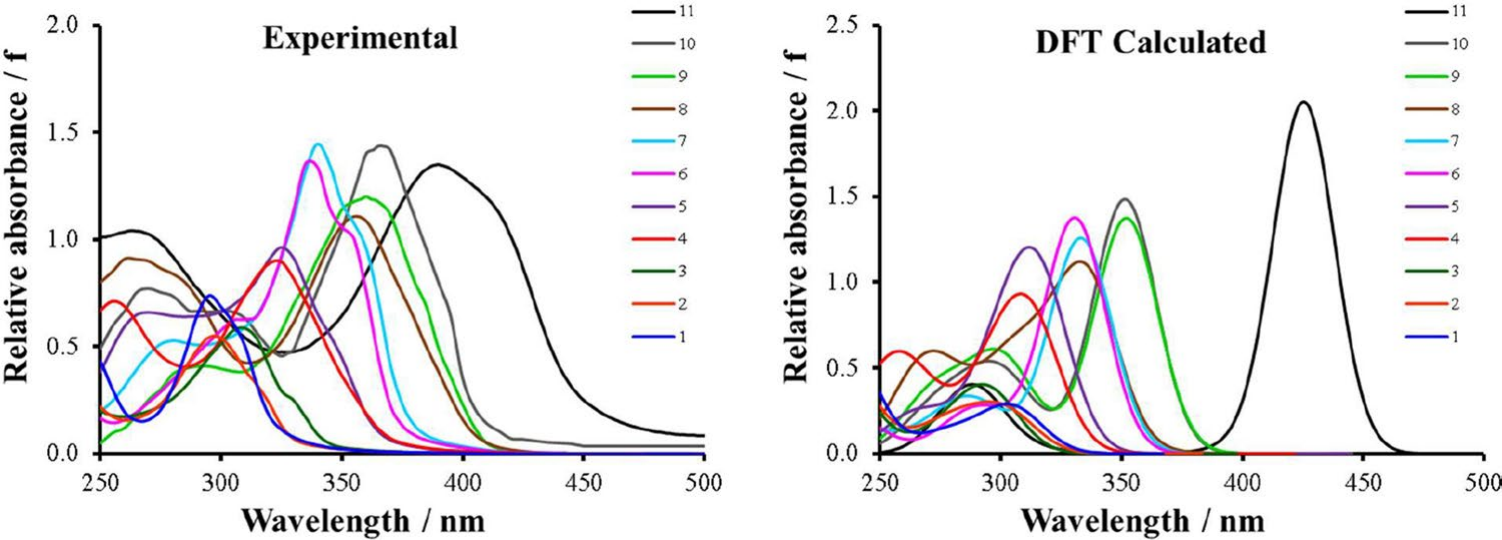
Ultraviolet/Visible spectra of **(1)**–**(11)**, in the 250–500 nm region. For calculated spectra, Gaussian broadening with FWHM = 30 in CH_3_CN was used. Experimental solvent as indicated in Table 1

Experimental and calculated ultraviolet/visible spectra of Cu(β-diketonato)_2_ complexes **(1)**–**(11)** in this study are given in Fig. 3, with the absorbance maxima (λ_A,max_) summarized in Table 1. The spectra of the Cu(β-diketonato)_2_ have a strong absorbance peak (λ_A,max_) in the 250–450 nm region. A good correlation is found between experimental and calculated spectra, with an average deviation (AD) of 13 nm. A bathochromic shift is observed for Cu(β-diketonato)_2_ complexes with no aromatic groups (**(1)**–**(3)**, 295–310 nm experimental), to complexes with one aromatic group (**(4)**–**(7)**, 325–340 nm experimental), to complexes with two aromatic groups (**(8)**–**(10)**, 360–363 nm experimental). This shift was observed for both the experimental and calculated complexes. The highest bathochromic shift was observed for Cu(di-tfth)_2_ (**(11)** 390 nm experimental), which contains an oligothiophene chain of two thiophene groups on one side of the β-diketone and a CF_3_ group on the other side. The complexes that were not experimentally analyzed in CH_3_CN also followed these trends that is also shown by the calculated spectra, all simulated in CH_3_CN.

**Table 1:** Experimental (solvent indicated) and TDDFT calculated wavelengths (in CH_3_CN), related to the absorbance maximum (λ_A,max_) in the UV\Vis region for **(1)**–**(11)**

**No**	**Complex**	**λ**_**A,max**_** (calc) (nm)**	**λ**_**A,max**_** (exp) (nm)**	**Difference calc-exp**^**a**^** (nm)**	**Exp solvent**	**Exp reference**
1	Cu(acac)_2_	304	295	9	CH_3_CN	[21]
2	Cu(tfaa)_2_	302	300	2	CH_3_CN	[21]
3	Cu(hfaa)_2_	295	310	15	CH_3_CN	[21]
4	Cu(ba)_2_	313	325	12	CH_3_CN	[21]
5	Cu(tfba)_2_	316	325	9	CH_3_CN	[21]
6	Cu(tffu)_2_	331	335	4	CH_3_CN	[21]
7	Cu(tfth)_2_	333	340	7	CH_3_CN	[21]
8	Cu(dbm)_2_	336	360	24	C_2_H_6_OS	[21]
9	Cu(thfph)_2_	352	362	10	CH_2_Cl_2_	[45]
10	Cu(dtm)_2_	352	363	11	CH_2_Cl_2_	[44]
11	Cu(di-tfth)_2_	425	390	35	CH_3_CN	[22]

^a^Average deviation (AD) from the difference between the calculated and experimental is 13 nm

By studying the charge transfer (CT) bands with the support of TDDFT (time-dependent density functional theory), more insight into this bathochromic shift can be obtained. In Table 2, the molecular orbitals (MOs) contributing to the maximum absorbance transitions are summarized. Complexes **(1)**–**(3)**, which have no aromatic groups on the β-diketone, involve excitation from occupied MOs that are ligand-based to MOs that are predominantly metal-based, hence, predominantly ligand-to-metal charge transfer (LMCT). These three complexes have 98% **(1)**, 95% **(2)** and 98% **(3)** orbital contributions from the HOMO-5 to the LUMO. The remainder of the complexes **(4)**–**(11)** involves excitation from occupied MOs that are ligand-based to MOs that are predominantly ligand-based, hence, predominantly ligand-to-ligand charge transfer (LLCT). For the metal component involved in the excitation of **(4)**–**(7)**, which have one aromatic group on the β-diketone, the orbital contributions to the metal-based LUMO show a decrease from 28 **(4)**, 8 **(5)** and 9 **(6)** to 7% **(7)**. The introduction of two aromatic groups in complex (**8**) splits the metal-based UMO between the LUMO and the LUMO + 4, with a total of 9% contribution to the *E*_λ__max_ excitation. For the remaining complexes **(9)**–**(11)**, the metal component remains on the LUMO + 4 and decreases from 3 **(9)** and 2 **(10)** to 1% **(11)** contribution to the *E*_λ__max_ excitation. As the metal component in the excited state character decreases, a bathochromic shift is observed. This is ascribed to the engagement of the aromatic substituent groups, amplifying the π-conjugations throughout the β-diketonato ligand leading to the LLCT bands.

**Table 2:** PBEh1PBE/aug-cc-pVDZ calculated energy (*E*_λmax_), absorbance maximum wavelength (λ_A,max_), oscillator strengths (*f*) and orbitals contributing to the transitions in the indicated excitation peaks of copper(II) complexes **(1)**–**(11)**

No	Complex	*E*_λ__max_	λ_A,max_	*f*	Orbital contributions			Character
		(eV)	(nm)		From	To	(%)	
**(1)**	Cu(acac)_2_	4.1	304	0.27	HOMO-5	LUMO	98	LMCT
**(2)**	Cu(tfaa)_2_	4.1	302	0.24	HOMO-5	LUMO	95	LMCT
**(3)**	Cu(hfaa)_2_	4.2	295	0.26	HOMO-5	LUMO	98	LMCT
**(4)**	Cu(ba)_2_	4.0	313	0.69	HOMO-3	LUMO + 2	6	LLCT
					HOMO-1	LUMO + 3	28	LLCT
					HOMO-17	LUMO	5	LMCT
					HOMO-12	LUMO	13	LMCT
					HOMO-11	LUMO	8	LMCT
					HOMO-6	LUMO	2	LMCT
					HOMO-2	LUMO + 2	4	LLCT
					HOMO	LUMO + 4	32	LLCT
**(5)**	Cu(tfba)_2_	3.9	316	0.86	HOMO-3	LUMO + 1	8	LLCT
					HOMO-1	LUMO + 3	35	LLCT
					HOMO-17	LUMO	4	LMCT
					HOMO-13	LUMO	4	LMCT
					HOMO-2	LUMO + 2	4	LLCT
					HOMO	LUMO + 4	44	LLCT
**(6)**	Cu(tffu)_2_	3.7	331	1.37	HOMO-3	LUMO + 1	25	LLCT
					HOMO-1	LUMO + 3	20	LLCT
					HOMO-17	LUMO	2	LMCT
					HOMO-9	LUMO	7	LMCT
					HOMO-2	LUMO + 2	21	LLCT
					HOMO	LUMO + 4	24	LLCT
**(7)**	Cu(tfth)_2_	3.7	333	1.22	HOMO-3	LUMO + 1	19	LLCT
					HOMO-1	LUMO + 3	26	LLCT
					HOMO-17	LUMO	2	LMCT
					HOMO-13	LUMO	5	LMCT
					HOMO-2	LUMO + 2	16	LLCT
					HOMO	LUMO + 4	31	LLCT
**(8)**	Cu(dbm)_2_	3.7	336	0.97	HOMO-3	LUMO + 1	8	LLCT
					HOMO-1	LUMO + 2	38	LLCT
					HOMO-25	LUMO	1	LMCT
					HOMO-25	LUMO + 4	2	LMCT
					HOMO-2	LUMO	3	LMCT
					HOMO-2	LUMO + 4	3	LMCT
					HOMO	LUMO + 3	43	LLCT
**(9)**	Cu(thfph)_2_	3.5	352	1.29	HOMO-4	LUMO + 2	2	LLCT
					HOMO-3	LUMO	16	LLCT
					HOMO-1	LUMO + 2	31	LLCT
					HOMO-25	LUMO + 4	2	LMCT
					HOMO-2	LUMO + 1	12	LLCT
					HOMO-2	LUMO + 4	1	LMCT
					HOMO	LUMO + 3	34	LLCT
**(10)**	Cu(dtm)_2_	3.5	352	1.42	HOMO-3	LUMO	16	LLCT
					HOMO-1	LUMO + 2	31	LLCT
					HOMO-25	LUMO + 4	2	LMCT
					HOMO-2	LUMO + 1	14	LLCT
					HOMO	LUMO + 3	35	LLCT
**(11)**	Cu(di-tfth)_2_	2.9	425	2.05	HOMO-3	LUMO	31	LLCT
					HOMO-2	LUMO + 2	18	LLCT
					HOMO-18	LUMO + 4	1	LMCT
					HOMO-1	LUMO + 1	29	LLCT
					HOMO	LUMO + 3	19	LLCT

### DSSC application

To be considered an effective dye in DSSCs, a complex should have a high molar extinction coefficient and absorb light efficiently over a broad range of the sunlight spectrum, particularly in the near ultraviolet and visible region (ca 300–800 nm). The experimental spectra for the Cu(II) complexes **(1)**–**(11)** are 295–390 nm, placing these complexes in the near UV range. The energies of the frontier MOs are crucial, as the HOMO and LUMO levels are associated with the molecule’s stability and reactivity. Furthermore, the LUMO and HOMO energies of a dye molecule should align favourably with the conduction band (CB) potential (*E*) of the semi-conductor in the DSSC (such as the TiO_2_ example in this study) and the redox potential (*E*_redox_) of the electrolyte (such as the I^−^/^3−^ example in this study) used in the DSSC [42]. To determine if the Cu(II) complexes in this study are suitable to be tested as dyes in DSSC, computationally derived properties of these prosperous dyes are summarized in Table 3.

**Table 3:** PBEh1PBE/aug-cc-pVDZ calculated values related to the indicated absorbance maximum excitation of Cu(II) complexes **(1)**–**(11)**. Calculations for a DSSC containing a ^−^/I^3−^ electrolyte and a TiO_2_ semi-conductor

No	Complex	*E*_λ__max_	λ_A,max_	*f*	LHE	τ	Δ*G*_inject_	Δ*G*_regenarate_	*E*_gap_	*V*_OC_^a^
		(eV)	(nm)			(ns)	(eV)	(eV)	(eV)	(V)
**(1)**	Cu(acac)_2_	4.1	304	0.27	0.467	5	1.4	1.9	4.5	1.9
**(2)**	Cu(tfaa)_2_	4.1	302	0.24	0.421	6	0.9	2.4	4.5	1.3
**(3)**	Cu(hfaa)_2_	4.2	295	0.26	0.453	5	0.4	3.0	4.5	0.7
**(4)**	Cu(ba)_2_	4.0	313	0.69	0.796	2	1.4	1.8	4.4	1.8
**(5)**	Cu(tfba)_2_	3.9	316	0.86	0.861	2	0.8	2.3	4.4	1.3
**(6)**	Cu(tffu)_2_	3.7	331	1.37	0.957	1	0.8	2.1	4.2	1.3
**(7)**	Cu(tfth)_2_	3.7	333	1.22	0.940	1	0.8	2.2	4.3	1.3
**(8)**	Cu(dbm)_2_	3.7	336	0.97	0.893	2	1.1	1.8	4.3	1.7
**(9)**	Cu(thfph)_2_	3.5	352	1.29	0.948	1	1.0	1.7	4.1	1.6
**(10)**	Cu(dtm)_2_	3.5	352	1.42	0.962	1	1.1	1.7	4.1	1.6
**(11)**	Cu(di-tfth)_2_	2.9	425	2.05	0.991	1	0.6	1.5	3.5	1.2

^a^Approximation from analytical relationship

The excitation energy (*E*_λmax_), which is correlated to the absorbance maximum wavelength (λ_A,max_), steadily decreases as the wavelength of the series **(1)**–**(11)** increases. A longer wavelength (such as **(11)**) is more favourable for DSSCs, since it is closer to the visible spectrum area. The LHE is correlated to *f*. Within the framework of DSSCs, LHE relates to the process of harnessing and transforming energy obtained from sunlight into electricity. This process is fundamental to the functioning of DSSCs, as it represents the first step where light energy is absorbed and transformed into electrical power. The photoactive dye is crucial in this process, as it absorbs and collects light, enabling the semiconductor to produce electricity (Fig. 1). The LHE contributes directly to the short-circuit current density (*J*_sc_) in DSSC; see Eq. 5 [31, 32, 46, 47]. It is a key parameter that represents the current density (current per unit area) generated by the DSSC when the cell’s terminals are shorted together, meaning there is no external voltage applied across the cell and is important for improving the performance of DSSCs.

5 $${J}_{\text{sc}}=\underset{\lambda }{{\int }}\text{LHE}\left(\lambda \right){\phi }_{\text{inject}}{\eta }_{\text{collect}}d\lambda$$

*η*_collect_ is the charge collection efficiency (generally constant for a specific DSSC). *ϕ*_inject_ is the electron injection efficiency, which is closely related to Δ*G*_inject_ calculated by Eq. (3) [8, 32, 46–49].

In this study, the complexes with no aromatic rings, **(1)**–**(3)**, with a maximum absorbance LMCT band, have a distinct lower LHE (0.451–0.467) than the remaining complexes, **(4)**–**(11)**, with maximum absorbance LLCT bands, that contains one or more aromatic rings on the β-diketonato ligand (0.796–0.991). The calculated excited state lifetime (τ) of the Cu(II) complexes in this study ranges from 1 to 6 ns. This is significantly lower than the values reported for the established dyes such as YD2-o-C8 (Zn–porphyrin type, 12 ns [50]) and CYC-B11 (Ru-bipyridine type, 27 ns [51]). The half-time for regeneration (the time required for the dye to recover its original state after undergoing a photo-induced reaction) of another established dye, *cis*-Ru(dcbpy)_2_(NCS)_2_, ranges from 100 ns to 10 µs [52]. This suggests that the excited state of the complexes could be long enough to adequately decelerate the charge recombination process necessary for an efficient DSSC.

**Fig. 4:**
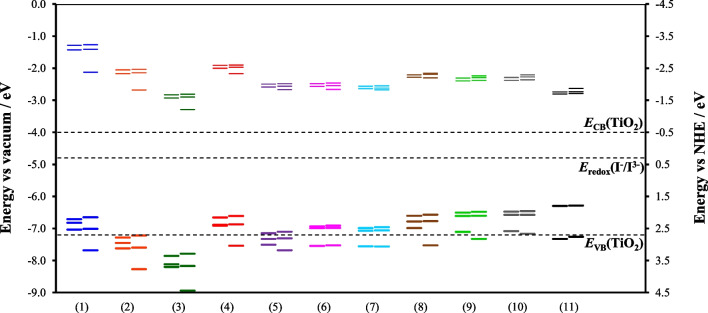
MO energy levels of Cu(β-diketonato)_2_ complexes **(1)**–**(11)**. For each complex, the left lines are alpha orbitals and the right lines are beta orbitals. The thicker bottom lines are OMO and the thinner top lines are UMO. Also shown: the charge band (CB) at − 4.0 eV and valence band (VB) at − 7.2 eV of TiO_2_ and redox potential at − 4.8 eV of the I^−^/I^3−^ electrolyte

In DSSCs, Δ*G*_inject_ and Δ*G*_regenerate_ are determined by comparing the calculated energies of *E*_HOMO_ and *E*_LUMO_ (or the higher energy UMOs involved in the excitation), with the conduction band potential of the semiconductor (*E*_CB_ =  − 4.0 eV relative to vacuum; − 0.5 eV relative to NHE for TiO_2_) and the redox potential of the electrolyte (− 4.8 eV relative to vacuum; 0.3 eV relative to NHE for the commonly used I^−^/I^3−^ electrolyte), as shown in Eqs. 3 and 4 and Fig. 4. A more negative *E*_HOMO_ relative to the redox potential of the electrolyte indicates a faster regeneration of oxidized dyes, while a higher *E*_LUMO_ compared to *E*_CB_ ensures efficient electron injection from the excited state. Analyzing Δ*G*_inject_ and Δ*G*_regenerate_ is vital in both experimental and theoretical research on redox mediators and dye sensitizers, as it helps to identify suitable candidates for DSSCs. Larger values of Δ*G*_inject_ and Δ*G*_regenerate_ facilitate easier charge transfer between the semiconductor’s conduction band and the electrolyte. For a DSSC to operate efficiently, Δ*G*_*i*nject_ should be greater than 0.2 eV [53, 54]. In this study, all complexes exhibit Δ*G*_inject_ values exceeding 0.2 eV, as shown in Table 3. The calculated energy gap (*E*_gap_) for complexes **(1)** to **(11)** decreases progressively with the addition of aromatic groups to the β-diketonato ligand, accompanied by an increase in the maximum absorbance wavelength. Given that the solar spectrum peaks around 500 nm (approximately 2.50 eV) [23], a smaller *E*_gap_ is beneficial for achieving larger photocurrents in DSSCs.

Figure 4 presents the energies of the alpha and beta MOs for **(1)**–**(11)**. The thicker bottom lines represent the occupied molecular orbitals (OMOs), while the thinner top lines indicate the unoccupied molecular orbitals (UMOs). These MO energies are associated with the conduction band and valence band potentials of the TiO_2_ semiconductor, as well as the redox potential (*E*_redox_) of the I^−^/I^3−^ electrolyte. All UMOs of compounds **(1)**–**(11)** exhibit higher energies than the conduction band edge of TiO_2_ (*E*_CB_(TiO_2_)), ensuring a sufficient driving force for electron injection. Additionally, the energies of the HOMOs of these complexes are below that of the redox potential of the I^−^/I^3−^ system, providing a substantial driving force for the regeneration of the dye.

*V*_oc_ represents the open-circuit voltage in a DSSC. It is a crucial parameter that represents the maximum voltage difference between the anode and cathode of the DSSC when no external circuit is connected (i.e. when the circuit is open and no current is flowing). *V*_oc_ is a measure of the energy conversion efficiency of the cell. It reflects the difference in energy between the Fermi level of the semiconductor and the redox potential of the electrolyte (*E*_CB_ − *E*_redox_) and can be estimated by Eq. (6) [30, 55–57].

6 $${\text{e}}{V}_{\text{oc}}\approx {E}_{\text{LUMO}}-{E}_{\text{CB}}$$

Improving *V*_oc_ is one of the strategies for enhancing the performance and efficiency of dye-sensitized solar cells. Equation (6) indicates that a more positive *E*_LUMO_ value enhances *V*_oc_, thus improving the energy conversion efficiency of the DSSC. From Table 3, *V*_oc_ for **(1)**–**(11)** are all above 1, with complexes **(1)**, **(4)** and **(8)** exhibiting the highest *V*_oc_ and complex **(3)** the lowest.

## Conclusions

The introduction of an aromatic ring at the groups on the β-diketonato ligand on Cu(β-diketonato)_2_ improved the DSSC properties of the complexes in this study by enhancing the oscillator strength, LHE and bathochromic shift of the maximum absorbance band into the visible region of the UV/vis spectrum. The frontier MOs (HOMO and LUMO) energies of complexes **(1)**–**(11)** are all favourable for electron injection into the TiO_2_ semiconductor and regeneration of the dye by the I^−^/I^3−^ electrolyte. With modification to a further bathochromic shift of the maximum absorbance band Cu(β-diketonato)_2_ complexes could be thought of as an eco-friendly and affordable dye for DSSCs.

## Supplementary Information

Below is the link to the electronic supplementary material.

Supplementary file1Optimized coordinates of the DFT calculations.(DOCX 197 KB)

## Data Availability

All data is provided within the article and supplementary information.
