# Supplementary material for: Cu(β-diketonato)2 bathochromic shifts from the ultraviolet towards the visible region
Source: J Mol Model. 2024 Sep 17;30(10):336. doi: 10.1007/s00894-024-06138-1 (PMC11408553; doi:10.1007/s00894-024-06138-1)
Supplement: Supplementary file 1 — Supplementary file1Optimized coordinates of the DFT calculations.(DOCX 197 KB) [file 894_2024_6138_MOESM1_ESM.docx]

Cu(β-diketonato)_2_ Bathochromic shifts from the Ultraviolet towards the Visible region

Supporting Information

Marrigje M Conradie*

Chemistry Department, University of the Free State, Bloemfontein, Republic of South Africa

Contact details:

Name: Marrigje M Conradie, Tel: +27-51-4019898, email: conradiemm@ufs.ac.za


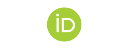
0000-0001-8104-7684 (MM Conradie)

***Optimized coordinates (xyz) of Cu(β-diketonato)_2_ complexes in this study.***

Cu(acac)_2_ (1)

Cu 0.000000000 0.000000000 0.000000000

O -1.637951000 1.010320000 -0.030271000

O 0.750985000 0.974452000 -1.482193000

C -1.088562000 2.434280000 -1.852560000

H -1.453292000 3.249257000 -2.472444000

C 1.002049000 2.515317000 -3.238267000

H 1.247203000 1.725951000 -3.961715000

H 1.951003000 2.893561000 -2.834568000

H 0.475414000 3.326603000 -3.749974000

C -1.926650000 1.957046000 -0.834557000

C -3.279651000 2.577555000 -0.636895000

H -3.350203000 2.970896000 0.386281000

H -4.050797000 1.802349000 -0.741842000

H -3.474691000 3.383582000 -1.350807000

C 0.190565000 1.925666000 -2.120818000

O 1.637951000 -1.010320000 0.030271000

O -0.750985000 -0.974452000 1.482193000

C 1.088562000 -2.434280000 1.852560000

H 1.453292000 -3.249257000 2.472444000

C -1.002049000 -2.515317000 3.238267000

H -1.247203000 -1.725951000 3.961715000

H -1.951003000 -2.893561000 2.834568000

H -0.475414000 -3.326603000 3.749974000

C 1.926650000 -1.957046000 0.834557000

C 3.279651000 -2.577555000 0.636895000

H 3.350203000 -2.970896000 -0.386281000

H 4.050797000 -1.802349000 0.741842000

H 3.474691000 -3.383582000 1.350807000

C -0.190565000 -1.925666000 2.120818000

Cu(tfaa)_2_ (2)

Cu 0.000000000 0.000000000 0.000000000

O -1.634492000 1.017991000 -0.037801000

O 0.754885000 0.970493000 -1.484471000

C -1.096841000 2.441651000 -1.857094000

H -1.449687000 3.254887000 -2.482455000

C 1.009208000 2.497062000 -3.225077000

C -1.940510000 1.959665000 -0.827363000

C -3.289615000 2.577698000 -0.628082000

H -3.359285000 2.960380000 0.398860000

H -4.057762000 1.800655000 -0.739421000

H -3.482815000 3.388159000 -1.336386000

C 0.158959000 1.912667000 -2.091122000

O 1.634492000 -1.017991000 0.037801000

O -0.754885000 -0.970493000 1.484471000

C 1.096841000 -2.441651000 1.857094000

H 1.449687000 -3.254887000 2.482455000

C -1.009208000 -2.497062000 3.225077000

C 1.940510000 -1.959665000 0.827363000

C 3.289615000 -2.577698000 0.628082000

H 3.359285000 -2.960380000 -0.398860000

H 4.057762000 -1.800655000 0.739421000

H 3.482815000 -3.388159000 1.336386000

C -0.158959000 -1.912667000 2.091122000

F -2.168054000 -2.982531000 2.741107000

F -1.318391000 -1.542077000 4.122142000

F -0.401600000 -3.492564000 3.889544000

F 0.401600000 3.492564000 -3.889544000

F 1.318391000 1.542077000 -4.122142000

F 2.168054000 2.982531000 -2.741107000

Cu(hfaa)_2_ (3)

Cu 0.000000000 0.000000000 0.000000000

O -1.636911000 1.019560000 -0.033403000

O 0.743758000 0.977939000 -1.487927000

C -1.097395000 2.450931000 -1.867331000

H -1.461026000 3.263208000 -2.485564000

C 1.026341000 2.499903000 -3.234232000

C -1.897802000 1.949532000 -0.841762000

C -3.286857000 2.564523000 -0.612155000

C 0.168627000 1.915605000 -2.101382000

O 1.636911000 -1.019560000 0.033403000

O -0.743758000 -0.977939000 1.487927000

C 1.097395000 -2.450931000 1.867331000

H 1.461026000 -3.263208000 2.485564000

C -1.026341000 -2.499903000 3.234232000

C 1.897802000 -1.949532000 0.841762000

C 3.286857000 -2.564523000 0.612155000

C -0.168627000 -1.915605000 2.101382000

F -3.570091000 3.549481000 -1.475061000

F -4.235354000 1.622230000 -0.736128000

F -3.369093000 3.067950000 0.630004000

F 2.170803000 2.995705000 -2.736058000

F 1.345344000 1.539642000 -4.116453000

F 0.408046000 3.483396000 -3.901659000

F 4.235354000 -1.622230000 0.736128000

F 3.369093000 -3.067950000 -0.630004000

F 3.570091000 -3.549481000 1.475061000

F -0.408046000 -3.483396000 3.901659000

F -2.170803000 -2.995705000 2.736058000

F -1.345344000 -1.539642000 4.116453000

Cu(ba)_2_ (4)

Cu 0.000000000 0.000000000 0.000000000

O 1.026419000 1.515552000 0.585843000

O 0.872600000 -0.052652000 -1.714034000

C 1.945071000 2.105323000 -0.074185000

C 2.354135000 1.769830000 -1.372458000

H 3.167116000 2.346934000 -1.800986000

C 1.797459000 0.724441000 -2.126416000

C 2.295710000 0.447607000 -3.504714000

C 2.986674000 1.406266000 -4.259718000

H 3.167006000 2.401809000 -3.856721000

C 3.424103000 1.108981000 -5.548137000

H 3.949918000 1.867228000 -6.128601000

C 3.185745000 -0.151940000 -6.096608000

H 3.532758000 -0.384365000 -7.103860000

C 2.497219000 -1.112117000 -5.353289000

H 2.307673000 -2.098630000 -5.777070000

C 2.048572000 -0.811476000 -4.070495000

H 1.504674000 -1.552576000 -3.487066000

C 2.615495000 3.244124000 0.639023000

H 1.859507000 3.992440000 0.912923000

H 3.391070000 3.717403000 0.029395000

H 3.060191000 2.873503000 1.572561000

O -1.026419000 -1.515552000 -0.585843000

C -1.945071000 -2.105323000 0.074185000

C -2.354135000 -1.769830000 1.372458000

H -3.167116000 -2.346934000 1.800986000

C -1.797459000 -0.724441000 2.126416000

O -0.872600000 0.052652000 1.714034000

C -2.295710000 -0.447607000 3.504714000

C -2.986674000 -1.406266000 4.259718000

H -3.167006000 -2.401809000 3.856721000

C -3.424103000 -1.108981000 5.548137000

H -3.949918000 -1.867228000 6.128601000

C -3.185745000 0.151940000 6.096608000

H -3.532758000 0.384365000 7.103860000

C -2.497219000 1.112117000 5.353289000

H -2.307673000 2.098630000 5.777070000

C -2.048572000 0.811476000 4.070495000

H -1.504674000 1.552576000 3.487066000

C -2.615495000 -3.244124000 -0.639023000

H -1.859507000 -3.992440000 -0.912923000

H -3.391070000 -3.717403000 -0.029395000

H -3.060191000 -2.873503000 -1.572561000

Cu(tfba)_2_ (5)

Cu -0.000523000 -0.000884000 -0.001595000

O 1.835512000 -0.572766000 0.013739000

O 0.484726000 1.852232000 -0.165150000

C 2.864063000 1.547171000 -0.181754000

H 3.793263000 2.099548000 -0.244395000

C 2.886539000 0.133415000 -0.063922000

C 1.685162000 2.265540000 -0.220962000

O -1.836557000 0.570997000 -0.016926000

O -0.485771000 -1.854001000 0.161954000

C -2.865108000 -1.548943000 0.178530000

H -3.794308000 -2.101322000 0.241155000

C -2.887585000 -0.135186000 0.060713000

C -1.686207000 -2.267311000 0.217748000

C 4.175150000 -0.604745000 -0.025002000

C 5.418986000 0.040797000 -0.101007000

C 4.141588000 -2.003278000 0.092549000

C 6.598870000 -0.696367000 -0.060246000

C 5.321116000 -2.737951000 0.133108000

C 6.553513000 -2.085999000 0.056770000

H 5.484103000 1.122871000 -0.192652000

H 3.176962000 -2.503542000 0.151461000

H 7.558335000 -0.183354000 -0.120133000

H 5.281385000 -3.823332000 0.224579000

H 7.479204000 -2.661194000 0.088485000

C -4.176197000 0.602972000 0.021781000

C -5.420033000 -0.042574000 0.097754000

C -4.142635000 2.001507000 -0.095751000

C -6.599917000 0.694589000 0.056982000

C -5.322163000 2.736178000 -0.136322000

C -6.554561000 2.084222000 -0.060015000

H -5.485149000 -1.124650000 0.189383000

H -3.178009000 2.501773000 -0.154640000

H -7.559382000 0.181572000 0.116844000

H -5.282434000 3.821560000 -0.227778000

H -7.480252000 2.659416000 -0.091739000

C 1.758782000 3.791216000 -0.349854000

C -1.759827000 -3.792988000 0.346628000

F -1.157604000 -4.383792000 -0.702861000

F -1.119394000 -4.203112000 1.457841000

F -3.014923000 -4.266050000 0.408382000

F 1.118334000 4.201333000 -1.461060000

F 3.013877000 4.264276000 -0.411627000

F 1.156575000 4.382027000 0.699640000

Cu(tffu)_2_ (6)

Cu 0.000026000 0.000012000 0.000145000

O -1.669879000 0.958121000 0.000160000

O -0.866875000 -1.720657000 0.000189000

C -3.128265000 -0.931591000 0.000236000

H -4.164707000 -1.249966000 0.000268000

C -2.519521000 -3.363250000 0.000252000

C -2.839151000 0.457060000 0.000204000

C -2.128359000 -1.882163000 0.000225000

O 1.669931000 -0.958097000 0.000137000

O 0.866927000 1.720681000 0.000104000

C 3.128316000 0.931615000 0.000095000

H 4.164758000 1.249990000 0.000080000

C 2.839202000 -0.457036000 0.000120000

C 2.128410000 1.882187000 0.000088000

F -3.846667000 -3.565778000 0.000309000

F -2.016664000 -3.983979000 1.084461000

F -2.016756000 -3.983994000 -1.083993000

C -3.944940000 2.782512000 0.000191000

C -5.310357000 3.170389000 0.000220000

C -6.030640000 2.006752000 0.000261000

O -5.214431000 0.936132000 0.000261000

C -3.932964000 1.408633000 0.000216000

H -3.069831000 3.422850000 0.000155000

H -5.715517000 4.176523000 0.000211000

H -7.094196000 1.796522000 0.000294000

C 2.519572000 3.363274000 0.000054000

F 3.846719000 3.565802000 0.000049000

F 2.016762000 3.984035000 1.084267000

F 2.016761000 3.983985000 -1.084187000

C 3.944992000 -2.782488000 0.000156000

C 5.310409000 -3.170365000 0.000155000

C 6.030691000 -2.006728000 0.000127000

O 5.214482000 -0.936108000 0.000113000

C 3.933016000 -1.408609000 0.000130000

H 3.069883000 -3.422826000 0.000174000

H 5.715568000 -4.176499000 0.000171000

H 7.094247000 -1.796497000 0.000116000

Cu(tfth)_2_ (7)

Cu 0.000000000 0.000000000 0.000009000

O 0.469856000 -1.865136000 0.000073000

O 1.841717000 0.564026000 0.000013000

C 2.850942000 -1.590818000 0.000102000

H 3.784073000 -2.142227000 0.000136000

C 1.666534000 -2.297410000 0.000106000

C 1.723969000 -3.828106000 0.000149000

C 2.880023000 -0.171261000 0.000054000

O -0.469856000 1.865136000 -0.000054000

O -1.841717000 -0.564026000 0.000004000

C -2.850942000 1.590818000 -0.000101000

C -1.666534000 2.297410000 -0.000097000

C -1.723969000 3.828106000 -0.000169000

C -2.880023000 0.171261000 -0.000048000

C -5.446962000 -0.012020000 -0.000092000

C -4.161197000 -0.520452000 -0.000051000

S -4.180458000 -2.260162000 0.000009000

C -5.895780000 -2.284241000 -0.000030000

C -6.437826000 -1.019995000 -0.000080000

H -6.419625000 -3.236747000 -0.000012000

H -7.508276000 -0.825757000 -0.000108000

F -2.974816000 4.316636000 -0.000188000

F -1.097708000 4.323834000 1.084023000

F -1.097719000 4.323726000 -1.084419000

F 1.097684000 -4.323752000 1.084365000

F 1.097743000 -4.323807000 -1.084077000

F 2.974816000 -4.316636000 0.000195000

C 6.437826000 1.019995000 0.000064000

C 5.895780000 2.284241000 0.000020000

S 4.180458000 2.260162000 -0.000006000

C 4.161197000 0.520452000 0.000050000

C 5.446962000 0.012020000 0.000081000

H 7.508276000 0.825757000 0.000085000

H 6.419625000 3.236747000 0.000000000

H 5.675465000 -1.051414000 0.000116000

H -5.675465000 1.051414000 -0.000130000

H -3.784073000 2.142227000 -0.000143000

Cu(dbm)_2_ (8)

Cu 0.000000000 0.000000000 0.000000000

O -1.666913000 0.948605000 -0.090580000

O 0.709527000 0.912957000 -1.533660000

C -1.143190000 2.344126000 -1.940600000

H -1.491519000 3.179968000 -2.535988000

C -1.977540000 1.872647000 -0.915242000

C 0.140514000 1.840436000 -2.201828000

O 1.666913000 -0.948605000 0.090580000

O -0.709527000 -0.912957000 1.533660000

C 1.143190000 -2.344126000 1.940600000

H 1.491519000 -3.179968000 2.535988000

C 1.977540000 -1.872647000 0.915242000

C -0.140514000 -1.840436000 2.201828000

C -3.328816000 2.472284000 -0.719645000

C -3.991891000 3.176864000 -1.734752000

C -3.965741000 2.307808000 0.518846000

C -5.259886000 3.708383000 -1.512847000

C -5.227886000 2.849562000 0.743654000

C -5.879406000 3.550905000 -0.272321000

H -3.533492000 3.294306000 -2.715555000

H -3.454017000 1.753971000 1.304128000

H -5.768938000 4.244042000 -2.314208000

H -5.707229000 2.723240000 1.714713000

H -6.870582000 3.971083000 -0.099167000

C -0.948769000 -2.408108000 3.318998000

C -2.338930000 -2.224236000 3.306047000

C -0.366270000 -3.101573000 4.389648000

C -3.130550000 -2.737227000 4.329382000

C -1.157890000 -3.603841000 5.419526000

C -2.542002000 -3.428000000 5.390063000

H -2.789698000 -1.678552000 2.478794000

H 0.713720000 -3.232511000 4.440338000

H -4.211354000 -2.596631000 4.301507000

H -0.691265000 -4.130996000 6.251762000

H -3.160723000 -3.825519000 6.195123000

C 3.328816000 -2.472284000 0.719645000

C 3.991891000 -3.176864000 1.734752000

C 3.965741000 -2.307808000 -0.518846000

C 5.259886000 -3.708383000 1.512847000

C 5.227886000 -2.849562000 -0.743654000

C 5.879406000 -3.550905000 0.272321000

H 3.533492000 -3.294306000 2.715555000

H 3.454017000 -1.753971000 -1.304128000

H 5.768938000 -4.244042000 2.314208000

H 5.707229000 -2.723240000 -1.714713000

H 6.870582000 -3.971083000 0.099167000

C 0.948769000 2.408108000 -3.318998000

C 2.338930000 2.224236000 -3.306047000

C 0.366270000 3.101573000 -4.389648000

C 3.130550000 2.737227000 -4.329382000

C 1.157890000 3.603841000 -5.419526000

C 2.542002000 3.428000000 -5.390063000

H 2.789698000 1.678552000 -2.478794000

H -0.713720000 3.232511000 -4.440338000

H 4.211354000 2.596631000 -4.301507000

H 0.691265000 4.130996000 -6.251762000

H 3.160723000 3.825519000 -6.195123000

Cu(thfph)_2_ (9)

Cu 0.000000000 0.000000000 0.000000000

O 0.697576000 -0.654771000 1.666132000

O 0.868933000 -1.379239000 -1.013916000

F 3.621961000 -5.704753000 -4.561432000

S 1.300438000 -0.875336000 4.457530000

C 2.154222000 -1.757348000 5.659850000

H 2.083655000 -1.455928000 6.701785000

C 2.867768000 -2.803166000 5.125940000

H 3.474455000 -3.482200000 5.721800000

C 2.721665000 -2.893269000 3.720074000

H 3.208377000 -3.656462000 3.116769000

C 1.899179000 -1.915461000 3.197969000

C 1.486747000 -1.650970000 1.816568000

C 1.965033000 -2.474258000 0.784211000

H 2.648767000 -3.271185000 1.053292000

C 1.627992000 -2.304865000 -0.565623000

C 2.173959000 -3.231333000 -1.597425000

C 2.759378000 -4.464705000 -1.275639000

H 2.824354000 -4.797677000 -0.241546000

C 3.247458000 -5.306518000 -2.269719000

H 3.694626000 -6.269908000 -2.030493000

C 3.146462000 -4.893192000 -3.590382000

C 2.572937000 -3.683315000 -3.954275000

H 2.513515000 -3.399463000 -5.003856000

C 2.082627000 -2.859947000 -2.946978000

H 1.621463000 -1.907478000 -3.200592000

O -0.697576000 0.654771000 -1.666132000

O -0.868933000 1.379239000 1.013916000

F -3.621961000 5.704753000 4.561432000

S -1.300438000 0.875336000 -4.457530000

C -2.154222000 1.757348000 -5.659850000

H -2.083655000 1.455928000 -6.701785000

C -2.867768000 2.803166000 -5.125940000

H -3.474455000 3.482200000 -5.721800000

C -2.721665000 2.893269000 -3.720074000

H -3.208377000 3.656462000 -3.116769000

C -1.899179000 1.915461000 -3.197969000

C -1.486747000 1.650970000 -1.816568000

C -1.965033000 2.474258000 -0.784211000

H -2.648767000 3.271185000 -1.053292000

C -1.627992000 2.304865000 0.565623000

C -2.173959000 3.231333000 1.597425000

C -2.759378000 4.464705000 1.275639000

H -2.824354000 4.797677000 0.241546000

C -3.247458000 5.306518000 2.269719000

H -3.694626000 6.269908000 2.030493000

C -3.146462000 4.893192000 3.590382000

C -2.572937000 3.683315000 3.954275000

H -2.513515000 3.399463000 5.003856000

C -2.082627000 2.859947000 2.946978000

H -1.621463000 1.907478000 3.200592000

Cu(dtm)_2_ (10)

Cu 4.840754000 3.836950000 11.531761000

O 6.576810000 3.414252000 10.820278000

O 3.971621000 3.551336000 9.844612000

S 9.400456000 2.959384000 10.633968000

C 10.701195000 2.513972000 9.603267000

H 11.711769000 2.465018000 10.000511000

H 10.951693000 1.959964000 7.517082000

H 8.361018000 2.266112000 7.226536000

C 10.280720000 2.257268000 8.320601000

C 8.882458000 2.423739000 8.168171000

C 8.252750000 2.805163000 9.335644000

C 6.840021000 3.079598000 9.613297000

C 5.897925000 2.963479000 8.578737000

H 6.254549000 2.671561000 7.597146000

C 4.526601000 3.203309000 8.745562000

C 3.615252000 3.054666000 7.591921000

C 2.259942000 3.276486000 7.711877000

H 1.719124000 3.569152000 8.606116000

C 3.976087000 2.680997000 6.255008000

H 4.987232000 2.457611000 5.923444000

C 2.904601000 2.630057000 5.411324000

H 2.887419000 2.378009000 4.354798000

S 1.435635000 3.036216000 6.229360000

O 3.104699000 4.259648000 12.243244000

O 5.709888000 4.122564000 13.218911000

S 0.281053000 4.714516000 12.429555000

C -1.019686000 5.159928000 13.460255000

H -2.030261000 5.208882000 13.063011000

C -0.599212000 5.416632000 14.742922000

H -1.270184000 5.713936000 15.546441000

C 0.799050000 5.250161000 14.895352000

H 1.320490000 5.407788000 15.836987000

C 1.428759000 4.868737000 13.727878000

C 2.841488000 4.594302000 13.450226000

C 3.783583000 4.710421000 14.484785000

H 3.426960000 5.002339000 15.466376000

C 5.154908000 4.470591000 14.317961000

C 6.066257000 4.619234000 15.471601000

C 7.421567000 4.397414000 15.351645000

H 7.962384000 4.104748000 14.457407000

C 5.705422000 4.992903000 16.808515000

H 4.694277000 5.216289000 17.140079000

C 6.776907000 5.043843000 17.652199000

H 6.794090000 5.295891000 18.708725000

S 8.245874000 4.637684000 16.834163000

Cu(di-tfth)_2_ (11)

Cu 0.000000000 0.000000000 0.000000000

O -0.411324000 1.879203000 -0.002145000

O 1.900179000 0.313573000 -0.004655000

C 1.843047000 2.694773000 -0.008457000

H 2.432929000 3.604288000 -0.011114000

C 0.468821000 2.799800000 -0.005753000

C -0.161154000 4.195247000 -0.006522000

C 2.503039000 1.436247000 -0.007610000

O 0.411324000 -1.879203000 0.002145000

O -1.900179000 -0.313573000 0.004655000

C -1.843047000 -2.694773000 0.008457000

C -0.468821000 -2.799801000 0.005753000

C 0.161154000 -4.195247000 0.006523000

C -2.503039000 -1.436247000 0.007610000

C -4.882372000 -2.412712000 0.015198000

C -3.952578000 -1.388127000 0.009615000

S -4.737821000 0.165978000 0.003860000

C -6.310031000 -0.570074000 0.012028000

C -6.211098000 -1.954078000 0.016320000

H -7.076756000 -2.613765000 0.024256000

F -0.740818000 -5.190457000 0.012488000

F 0.938802000 -4.363562000 -1.080408000

F 0.947618000 -4.358540000 1.087801000

F -0.947618000 4.358540000 -1.087801000

F -0.938802000 4.363562000 1.080409000

F 0.740818000 5.190457000 -0.012488000

C 6.211098000 1.954078000 -0.016320000

C 6.310031000 0.570074000 -0.012029000

S 4.737821000 -0.165978000 -0.003859000

C 3.952578000 1.388127000 -0.009614000

C 4.882372000 2.412712000 -0.015198000

H 7.076756000 2.613765000 -0.024256000

H 4.616458000 3.467353000 -0.019898000

H -4.616458000 -3.467353000 0.019899000

H -2.432929000 -3.604288000 0.011114000

C -7.614954000 1.617655000 0.063293000

C -7.504251000 0.242479000 0.012494000

S -9.085630000 -0.490979000 -0.058438000

C -9.862761000 1.045335000 -0.017563000

C -8.957392000 2.073243000 0.046403000

H -6.753794000 2.282328000 0.115085000

H -9.245441000 3.122053000 0.080956000

C 8.957392000 -2.073243000 -0.046404000

C 9.862761000 -1.045335000 0.017562000

S 9.085630000 0.490979000 0.058436000

C 7.504251000 -0.242479000 -0.012495000

C 7.614954000 -1.617654000 -0.063294000

H 9.245441000 -3.122053000 -0.080957000

H 10.947812000 -1.097399000 0.043446000

H 6.753794000 -2.282328000 -0.115085000

H -10.947812000 1.097399000 -0.043448000
